# Low urine pH and acid excretion do not predict bone fractures or the loss of bone mineral density: a prospective cohort study

**DOI:** 10.1186/1471-2474-11-88

**Published:** 2010-05-10

**Authors:** Tanis R Fenton, Misha Eliasziw, Suzanne C Tough, Andrew W Lyon, Jacques P Brown, David A Hanley

**Affiliations:** 1 Department of Community Health Sciences, Faculty of Medicine, University of Calgary TRW Building, 3rd Floor, 3280 Hospital Drive NW, Calgary, Alberta, T2N 4Z6, Canada; 2 Alberta Health Services, Calgary, AB, Canada; 3 Department of Pathology & Laboratory Medicine, Faculty of Medicine, University of Calgary 9, 3535 Research Road NW, Calgary, AB, T2L 2K8, Canada; 4 Division of Rheumatology, Centre de recherche du CHUL, Université Laval, Quebec City, Canada; 5 Department of Medicine and Oncology, Faculty of Medicine, University of Calgary Foothills Medical Centre - North Tower, 9th Floor, 1403 - 29th Street NW, Calgary, AB T2N 2T9, Canada

## Abstract

**Background:**

The acid-ash hypothesis, the alkaline diet, and related products are marketed to the general public. Websites, lay literature, and direct mail marketing encourage people to measure their urine pH to assess their health status and their risk of osteoporosis.

The objectives of this study were to determine whether 1) low urine pH, or 2) acid excretion in urine [sulfate + chloride + 1.8x phosphate + organic acids] minus [sodium + potassium + 2x calcium + 2x magnesium mEq] in fasting morning urine predict: a) fragility fractures; and b) five-year change of bone mineral density (BMD) in adults.

**Methods:**

Design: Cohort study: the prospective population-based Canadian Multicentre Osteoporosis Study. Multiple logistic regression was used to examine associations between acid excretion (urine pH and urine acid excretion) in fasting morning with the incidence of fractures (6804 person years). Multiple linear regression was used to examine associations between acid excretion with changes in BMD over 5-years at three sites: lumbar spine, femoral neck, and total hip (n = 651). Potential confounders controlled included: age, gender, family history of osteoporosis, physical activity, smoking, calcium intake, vitamin D status, estrogen status, medications, renal function, urine creatinine, body mass index, and change of body mass index.

**Results:**

There were no associations between either urine pH or acid excretion and either the incidence of fractures or change of BMD after adjustment for confounders.

**Conclusion:**

Urine pH and urine acid excretion do not predict osteoporosis risk.

## Background

Osteoporosis is a disease that causes pain, disability, reduced quality of life [1], mortality [2], and places substantial demands on health care budgets [3,4]. According to the acid-ash hypothesis, the modern diet produces residual acid after metabolism [5-7]. This diet-derived acid is thought to be buffered by bicarbonate from bone, followed by bone calcium excretion in the urine [5-7].

Numerous papers in the medical literature (experimental trials [6-14], cross sectional studies [5,15], prospective studies [16-19], and animal models [20]) identify the potential acid load of the diet as a risk factor for osteoporosis. Well-respected textbooks [21] and reference works [22,23] uphold this concept. Of public health importance, the acid-ash hypothesis is marketed to the general public as the "alkaline diet", to decrease acidity, to help the body regulate its pH, and to prevent numerous disease processes. Websites[24,25], lay literature [26-28], magazine advertisements, and direct mail marketing encourage people to measure their urine pH to assess their risk of osteoporosis as well as their general health status [24,29]. Urine pH of people consuming modern diets tends to be slightly acidic, with pH of approximately 6 [14,30]. When urine pH is found to be acidic, the "alkaline diet" and the purchase of products to achieve acid-base balance are advocated.

In the medical literature, the diet acid load has been quantified in food ([sulfate + chloride + 1.8x phosphate + organic acids] minus [sodium + potassium + 2x calcium + 2x magnesium] in mEq/day [10]), as well as in urine [10,11,31]. The urine acid load has been quantified in two ways: through the measurement of urine pH [10,11,31], and from the measurement of net acid excretion [10,31-33]. Traditionally the urine acid excretion has been estimated from measured titratable acidity, ammonium and bicarbonate [10,32], which may be inferior measures of acid excretion since ammonium and bicarbonate are volatile and altered by bacterial growth [34] and the measurement of titratable acidity is not a precise method [35]. Urine measures of food intake or the diet acid load take place after absorption has occurred, are not prone to food intake reporting bias, and therefore may provide more accurate estimates.

To our knowledge, the utility of urine pH and/or urine measures of acid excretion to predict osteoporotic outcomes has not been assessed. If urine pH and urine measures of the potential acid load of the diet can predict which individuals are vulnerable to the development of osteoporosis, a novel laboratory testing strategy could be implemented to identify individuals at risk of accelerated bone loss. However, if these measures do not predict which individuals are vulnerable to osteoporosis, the practice of urine pH testing to identify who is at risk for this disease should be discouraged.

The objectives of this study were to determine whether 1) low urine pH, or 2) the calculated acid excretion in urine predict: a) fragility fractures; and b) five-year change of bone mineral density (BMD) in adults.

## Methods

**Figure 1:**
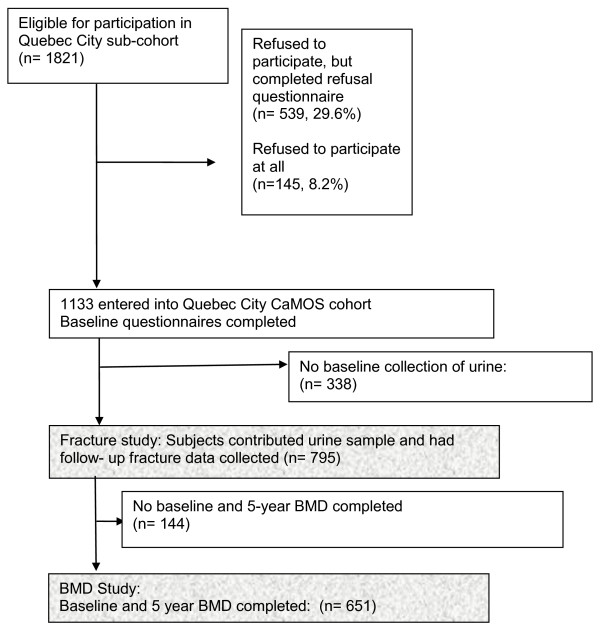
**Flow diagram of the entry of subjects into the fracture and BMD studies**.

This study was approved by the Conjoint Health Research Ethics Board, University of Calgary and the Canadian Multicentre Osteoporosis Study (CaMOS) Design, Analysis and Publication Committee. Informed consent was obtained from the study participants at baseline. CaMOS is a prospective population-based cohort study designed to study skeletal health among a random sample of Canadian adults 25 years of age and above [36].The participants in this study included those recruited from Quebec because they had urine and blood samples obtained at both baseline and at 5-years (Figure 1). At baseline (1996-8) and at five years participants had in-person interviews, measurement of BMD, and collection of samples. Fasting urine samples were collected in the morning after an initial void and a wait of two hours, while participants maintained a fast from the evening before. The urine samples were aliquoted, frozen and stored at -70°C in plastic vials within two hours of collection.

The outcome variables for this study were change of BMD (Dual Energy X-ray Absorptiometry (Hologic 2000)) over 5-years (at the femoral neck (FN), the lumbar spine (L1-4) (LS), and the total hip (HIP)) as well as fragility fractures over seven years of follow-up. Fractures, initially self-reported, were confirmed using radiology reports, over seven years of follow-up. A fragility fracture occurred spontaneously, after a no-fall injury, or from a fall from a standing height or less.

The exposure variables used in this study were: urine pH, and the concentration of the urine ions (mmol/l) summed as estimated acid excretion ([sulfate + chloride + 1.8x phosphate + estimated organic acids [37]] - [potassium + sodium + 2x calcium + 2xmagnesium] mEq/L) [10] based on measurements made on in fasting morning urine. Urine creatinine was included in the adjusted models to control for urine concentration. The urinary excretion of organic acids (including citric, oxalic, malic, succinic, lactic, glutamic, aspartic acid, etc) were estimated from body surface area ([(body surface area × 41)/1.73] [37], where 41 is the median daily organic acid anion excretion (mEq/d) (body surface area was used by these authors as [0.007184x *height *(cm)^0.725 ^× *weight *(kg)^0.425^] [37] based on a recent study that found that anthropometric measures predicted urine organic acids (R^2 ^of 0.15 to 0.39) better than diet measures [37].

Variables considered to be potential confounders included: age, gender, reported family history of osteoporosis among first degree relatives, body mass index (BMI), change in BMI [38-40], hormonal status among the women, presence or absence of kidney disease [41], smoking, medications (thiazide diuretics, bisphosphonates, and estrogen) use, physical activity [42], calcium intake, and vitamin D status. Vitamin D status was assigned as adequate if the serum 25-hydroxy vitamin D was >= 80 nmol/l [43] (Table 1). Medication use was based on prescription bottles viewed at baseline. Women were coded at baseline as estrogen sufficient if they were either premenopausal, or taking estrogen. Urine creatinine was included in the adjusted models to control for urine concentration. Baseline BMD was included in the adjusted models to control for the potential influence of baseline BMD on the change over time [44].

**Table 1:** Baseline subject characteristics in the fracture and the BMD studies

Subject characteristics	Fracture study	BMD study
n	795	651
Age	59.8 +/- 12.8	58.6 +/- 12.3
Female gender, (%)	553/795 (69.6%)	455/651 (69.9%)
Women estrogen sufficient, (%)	261/553 (47.2%)	228/455 (50.1%)
Body Mass Index, kg/m2	26.1 +/- 4.7	26.0 +/- 4.7
Change of BMI, kg/m2	0.56 +/- 1.71	0.59 +/- 1.69
Physical activity, kcal/day	1548 +/- 589	1576 +/- 806
Family history of osteoporosis, (%)	71/511 (13.9%)	60/415 (14.5%)
Calcium intake, mg/day	889 +/- 550	885 +/- 551
Serum 25(OH) Vitamin D, nmol/l	75.1 +/- 28.4	74.9 +/- 28.4
Smoking, (%)	154/438 (35.2%)	113/352 (32.1%)
Caucasian race, (%)	792 (99.6%)	647 (99.4%)
Kidney Disease, (%)	119/733 (16.2%)	118/632 (18.7%)
Thiazide medications, (%)	66/795 (8.3%)	52/651 (8.0%)
Bisphosphonates, (%)	8/795 (1.0%)	6/651 (0.9%)

*mean +/- sd, nmol/l = nanomole per litre

Immediately after thawing, urine pH was measured using a Radiometer PHM82 Standard pH Meter (Copenhagen, Denmark). The calcium, phosphate and creatinine measurements were made using a Vitros 950 (Vitros Chemistry product, Ortho-Clinical Diagnostics, Johnson and Johnson Company, Rochester, NY, USA. Sodium, potassium, magnesium, and chloride were measured using Cobas Integra 700 analyzer (Roche Diagnostics, F.Hoffmann-La Roche Ltd, Basel, Switzerland). Urine sulfate was measured using an automated methylthymol blue method.

### Regression analysis methods

Multiple logistic regression analysis was used for the fractures analysis and linear regression analysis was used for the BMD analysis. To determine the independent influence of urine pH, models were fully adjusted for potential confounders. Missing values were frequent for smoking status (44.9%), osteoporosis family history (26.8%) and serum 25(OH) vitamin D (49%). Rather than excluding subjects because they had missing values, a third category was added to each of these variables to assess the effect of missingness on outcome. As a result, two indicator variables were created to represent the 3 categories in the regression analyses for each of these variables. Subsequent to performing logistic regression analyses, the predictive accuracies of urine pH and urine acid excretion for fractures was quantified by the c-statistic, having values range from 0.5 (chance accuracy) to 1.0 (perfect accuracy), with the following intermediate benchmarks: 0.6 (fair), 0.7 (good), 0.8 (excellent), and 0.9 (almost perfect). Stata, Version 10.1 (StataCorp, College Station, Texas, USA), was used for the data analysis.

## Results

**Figure 2:**
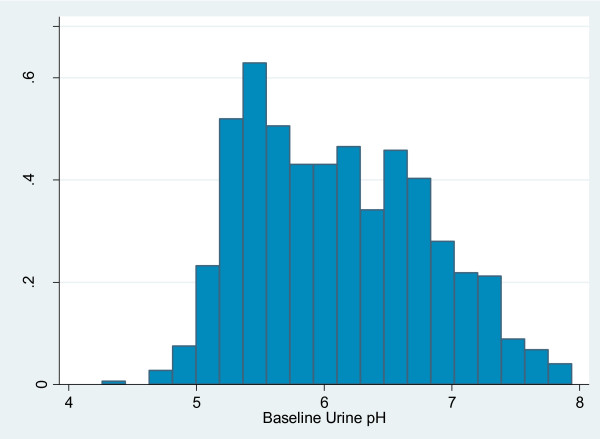
**Distribution of urine pH**.

The baseline questionnaire was completed by 1133 subjects (Figure 1); 795 contributed baseline urine samples, 651 (81.9%) had two BMD measurements. The distribution of urine pH is shown in Figure 2. By year seven complete fracture data was recorded for 6804 person years among the 795 subjects. The baseline characteristics and subjects' urine composition in the Fracture and BMD studies were similar (Table 1 &2).

**Table 2:** Urine values at baseline

Urine values	Fracture study	BMD study
Urine pH	6.1 +/- 0.70	6.1 +/- 0.71
Calcium/creatinine, mmol/mmol	0.37 +/- 0.24	0.38 +/- 0.24
Chloride/creatinine, mmol/mmol	17.4 +/- 9.0	17.1 +/- 8.7
Magnesium/creatinine, mmol/mmol	0.43 +/- 0.20	0.43 +/- 0.20
Phosphate/creatinine, mmol/mmol	2.51 +/-0.84	2.53 +/-0.84
Potassium/creatinine, mmol/mmol	6.18 +/- 3.05	6.14 +/- 3.07
Sodium/creatinine, mmol/mmol	16.4 +/- 9.2	16.1 +/- 8.9
Sulfate/creatinine, mmol/mmol	2.09 +/- 0.85	2.08 +/- 0.85
Organic acids/creatinine, mmol/mmol	6.89 +/- 5.37	6.76 +/- 5.25
Urine creatinine, mmol/L	8.8 +/- 5.2	8.9 +/- 5.3
Acid excretion, mEq/L	46.6 +/- 25.0	47.5 +/- 25.4

*mean +/- sd, mmol = millimole, L = litre, mEq/L = milliequivalent/litre

During follow-up, the mean FN BMD decreased significantly over 5-years by -0.68% (p < 0.001); the HIP BMD did not change significantly (-0.11%) (p = 0.66); and the LS increased significantly (1.92%) (p < 0.001) (Table 3). Eighty-seven (10.9%) cohort subjects had 112 fractures; 46 (5.8%) subjects sustained confirmed fragility fractures. Of those who sustained confirmed fragility fractures, 41 (89%) were aged 50 or greater.

**Table 3:** Bone outcomes in the Fracture and BMD studies

	Fracture study	BMD study
Bone Mineral Density (BMD):		
Baseline FN BMD, g/cm^2^	0.727 +/- 0.128	0.731 +/- 0.126
Baseline LS BMD, g/cm^2^	0.936 +/- 0.166	0.938 +/- 0.160
Baseline HIP BMD, g/cm^2^	0.880 +/- 0.151	0.885 +/- 0.146
Change BMD, over 5-years		
5-year change FN BMD, g/cm^2^	n/a	-0.005 +/- 0.035
5-year change LS BMD, g/cm^2^	n/a	0.018 +/- 0.054
5-year change HIP BMD, g/cm^2^	n/a	-0.001 +/- 0.036
Change FN BMD, % of baseline	n/a	-0.68% +/- 5.0
Change LS BMD, % of baseline	n/a	1.9% +/- 6.0
Change HIP BMD, % of baseline	n/a	-0.11% +/- 4.2
Confirmed fragility fractures	46/795 (5.8%)	n/a

*mean +/- sd, dichotomous variables are reported with (percentage), g/cm^2 ^= gram per squared centimetres

### Regression analysis results

There was no association between urine pH or urine acid excretion and the occurrence of fragility fractures (Table 4). Subjects had similar risks of fracture for those with low and high urine pH, whether or not potential confounders were controlled, since the odds ratios were all close to one. The c-statistics illustrated that the unadjusted models for both urine pH and urine acid excretion did not discriminate any better than chance, while the adjusted models had a good ability to predict who would have a fracture (Table 4).

**Table 4:** Multiple logistic regression analysis results: Urine pH and acid excretion associations with fragility fractures*

**Fracture type**		**Unadjusted estimate**	**p-value**	**Adjusted estimate**	**p-value**
Urine pH:					
Fragility fractures	Odds ratio:	1.07	0.76	0.97	0.91
	95% confidence interval:	0.70 to 1.63		0.58 to 1.63	
	c-statistic:	0.514		0.731	
Urine Acid excretion:					
Fragility fractures	Odds ratio:	1.00	0.76	1.00	0.92
	95% confidence interval:	0.99 to 1.01		0.99 to 1.02	
	c-statistic:	0.510		0.731	

*The following variables were included in the adjusted models to control for confounding: age, gender, family history of osteoporosis, body mass index (BMI), change in BMI, estrogen status, physical activity, calcium intake, vitamin D status, smoking, kidney disease, urine creatinine, thiazide diuretics, and bisphosphonate use.

There were two statistically significant associations among the unadjusted relationships between urine measures of acid excretion and change of BMD (Table 5): urine acid excretion with both the change of BMD at the FN and the LS (p = 0.026 and p = 0.041, respectively). Both the slope coefficients and adjusted R^2 ^were close to zero, indicating poor associations. Once adjusted for potential confounding variables, there was no relationship between urine pH or urine acid excretion with the five-year change of BMD for any of the three sites assessed, FN, LS, or HIP (Table 5). Specifically, in the unadjusted models, two relationships were statistically significant: urine acid excretion with both the change of BMD at the FN and the LS (p = 0.026 and p = 0.041, respectively). However, no relationships were statistically significant once confounding was controlled. Once adjusted for confounding, individuals' BMD equally increased and decreased for those with high and low urine pH, and for those with high and low acid excretion. The greatest amount of explained variance (adjusted R^2^) for the adjusted models for each bone site ranged from two to ten percent. Post estimation residual analysis did not identify any concerns with the assumptions of linear regression.

**Table 5:** Multiple linear regression analysis results: Urine pH and acid excretion associations with change of BMD*

**Bone Site:**		**Unadjusted estimate**	**p-value**	**Adjusted estimate**	**p-value**
Urine pH:					
Femoral Neck	Coefficient: 95% confidence interval: aR^2 ^:	0.0034 -0.0004 to 0.0073 0.003	0.08	0.0018 -0.0025 to 0.0062 0.086	0.41
Total hip	Coefficient: 95% confidence interval: aR^2 ^:	0.0012 -0.0027 to 0.0051 0.001	0.54	0.0020 -0.0020 to 0.0066 0.096	0.29
Lumbar Spine	Coefficient: 95% confidence interval: aR^2 ^:	0.0053 -0.0052 to 0.0111 0.003	0.07	0.0022 -0.0044 to 0.0089 0.026	0.51
Urine Acid excretion:					
Femoral Neck	Coefficient: 95% confidence interval: aR^2 ^:	-0.0001 -0.0002 to -0.00002 0.0062	0.026	-0.0001 -0.0002 to 0.00001 0.088	0.19
Total hip	Coefficient: 95% confidence interval: aR^2 ^:	-0.0001 -0.0002 to 0.00005 0.001	0.26	-0.0001 -0.0002 to 0.0001 0.099	0.46
Lumbar Spine	Coefficient: 95% confidence interval: aR^2 ^:	-0.0002 -0.0003 to -0.00001 0.005	0.041	-0.00002 -0.0002 to 0.0002 0.026	0.84

*The following variables were included in the adjusted models to control for confounding: age, gender, family history of osteoporosis, body mass index (BMI), change in BMI, estrogen status, physical activity, calcium intake, vitamin D status, smoking, kidney disease, urine creatinine, thiazide diuretics, bisphosphonate use and baseline BMD.

## Discussion

This study revealed no significant associations between either of the urine measures of dietary acid load, urine pH or calculated acid excretion in urine [10], with the occurrence of fragility fractures over 7-years or changes in BMD over 5-years at any of three sites (lumbar spine, femoral neck, or total hip). The adjusted models are more likely to be accurate estimations of the associations between dietary acid load in fasting morning urine samples and bone outcomes than the unadjusted models since the adjusted models control for other risk factors and confounding [45].

The evidence to support the acid-ash hypothesis of osteoporosis predominantly comes from studies using changes in urine calcium as the outcome [6,8-12,46], some prospective observational studies [16,18,19,47] and one randomized trial [13]. Although one randomized trial has supported the hypothesis [13], another did not [40]. The randomized trial [13] supporting the hypothesis did not use concealment of allocation, a study quality indicator for randomized studies that is important to avoid bias during the randomization process [48]. Without concealment of allocation, investigators can influence the allocation of individuals into the treatment groups, invalidating the randomization. Trials that do not have concealed allocation can overestimate effectiveness of a therapy [49]. The more recent randomized trial, that concealed allocation, did not reveal any protective effect of either potassium citrate or increased fruit and vegetable consumption on change in BMD [14].

Three previous prospective cohort studies of the hypothesis reported some protective associations between fruit and vegetable, potassium, and/or vitamin C intakes and bone health [16,18,19], and thus may support the hypothesis, while two more recent cohort studies do not support it [50,51]. One of the positive reporting studies did not see a significant protective association for potassium once potential confounders were controlled [16]. Further, it is possible that the associations in agreement with the hypothesis in the cohort studies were due to uncontrolled confounding by estrogen status [19], baseline BMD [16,19], change of weight status during follow-up [18], and/or vitamin D status [16,18,19,47].

Further, three recent meta-analyses of the acid-ash hypothesis do not support the hypothesis. First, a meta-analysis of calcium balance studies, restricted to studies of superior methodology, revealed no association between net acid excretion and calcium balance, in spite of the strong relationship between net acid excretion and urinary calcium [52]. A systematic review and meta-analysis of the effect of protein intake on bone health revealed a small beneficial effect of protein supplementation on lumbar spine BMD in randomized placebo-controlled trials [53]. Further, the acid-ash hypothesis predicts higher phosphate intakes would be associated with increased urinary calcium and lower calcium balance, but this was not supported by a third recent meta-analysis [54].

We used urine samples that had been stored since baseline. Urine pH may change slightly with storage as carbon dioxide (CO_2_) and ammonia (NH_3_) evaporate, but the changes are not likely to result in misrepresentation of the measurements. As urine bicarbonate evaporates as CO_2 _from alkaline urine (H^+ ^and HCO_3_^- ^<--> H_2_O + CO_2_), pH would increase through the loss of the hydrogen ions [55]. As urine ammonia (NH_3_) evaporates from more acidic urine, ammonium would converted to ammonia (NH_4_^+ ^<--> H^+ ^+ NH_3_), hydrogen ions would be liberated and therefore the measured hydrogen ion will increase and thus the pH will decrease. Bicarbonate is found in appreciable amounts only in more alkaline urine, and ammonium in more acidic urine [56]. Therefore these reactions, which cause the alkaline urine to become somewhat more alkaline, and acidic urine to become more acidic, would not likely cause misrepresentation of the measurements of the potential risks.

Our study had four strengths. First, the population from which the cohort was selected has wide ranges of intakes for protein, potassium, fruit and vegetables [57,58]. Therefore the sample likely had a range of exposures, which improve the chances of revealing the relationship under investigation. Second, the outcome measures used in this study, change in BMD (an accepted clinical measure of the progression of this disease [59]) and occurrence of fragility fractures (a direct measure of osteoporosis [60,61]) are superior measures of osteoporosis relative to the measurement of urine calcium changes. Third, the use of urine to measure the diet acid load allowed us to avoid the random and systematic errors inherent in food intake measurement. Food-based calculations of acid load excretion are not very precise [31], determining food intake is prone to reporting bias [62,63], and the absorption of individual ions from food is variable [10,64]. Food is a complex mixture of numerous compounds, and nutrient absorption depends on the chemical composition as well as "on various interactions with many endogenous and exogenous factors" [35].

This study also had limitations. First, the exposures for this study were measured in fasting morning urine samples, which might be inferior to measures made in 24-hour urine samples. It is possible that urine pH or measures of acid excretion in 24-hour urine samples have a relationship with bone outcomes, and it is also possible that fasting-morning-second-void urines are more reliable measures of diet-acid than 24-hour collections that are more prone to incomplete collection [65]. However, proponents of the acid-ash hypothesis advocate the measurement of pH in morning urine to assess osteoporosis risk [24,27], so we argue the ability to predict osteoporosis in morning urine samples warranted testing. Second, as a cohort study, the risk of confounding remains, and there is a chance of reporting bias for the potential confounding variables.

## Conclusion

This study indicates that two measures of acid excretion from fasting morning urine, low urine pH and the calculated acid excretion in urine, do not identify individuals at risk for osteoporosis, as measured using fragility fractures and changes of bone mineral density. This study cannot determine whether the lack of association between these urine measures of diet acid load and bone outcomes is due to the type of urine sample used, the lack of ability of a single measure to reflect long-term diet acid load, or because the acid-ash hypothesis is not predictive of bone health. Together, this study, the recent critical review [66], and recent meta-analyses [52-54] provide a body of contradicting evidence for the acid-ash hypothesis. Further research is needed to improve our understanding of the role of diet in prevention of osteoporosis and to provide the best possible evidence to support or refute marketing of novel therapies to influence health.

## Competing interests

The authors declare that they have no competing interests.

## Authors' contributions

The author's responsibilities were as follows: All of the authors designed the study, TRF performed the statistical analysis and wrote the manuscript, ME directed the study's statistical analysis, AWL contributed to data analysis and writing of manuscript, SCT, DAH and JPB helped design the study and interpret the findings. We thank Sue Ross for assistance with editing. The funding sources had no influence on the interpretation of results. None of the authors had a personal or financial conflict of interest.

## Pre-publication history

The pre-publication history for this paper can be accessed here:

http://www.biomedcentral.com/1471-2474/11/88/prepub
